# Aortic Regurgitation: An Updated Review of Etiologies, Diagnostic Strategies, and Clinical Management

**DOI:** 10.31083/RCM37259

**Published:** 2025-06-26

**Authors:** Randeep Gill, Noyan Ramazani, Michael V. DiCaro, KaChon Lei, Sigurd Hartnett, Tillmann Cyrus, Tahir Tak

**Affiliations:** ^1^Department of Internal Medicine, Kirk Kerkorian School of Medicine at UNLV, Las Vegas, NV 89102, USA; ^2^VA Southern Nevada Healthcare System, North Las Vegas, NV 89086, USA

**Keywords:** aortic regurgitation, imaging, echocardiography, magnetic resonance, computed tomography, aortography, valve replacement, surveillance

## Abstract

Aortic regurgitation is a valvular disorder that necessitates the integration of multiple aspects of clinical practice. The underlying etiologies span an array of pathologies, including congenital, infectious, structural, and traumatic causes. Imaging studies range from traditional cardiac diagnostic strategies to advanced imaging modalities. Meanwhile, depending on clinical presentation, management may involve a medical or surgical approach. Long-term surveillance and chronic disease management are crucial in preventing progression into further complications, such as heart failure. This review aims to provide a thorough, comprehensive analysis of the contemporary understanding of aortic regurgitation. The information we have included will offer a unique perspective on how recent updates in diagnostic and management strategies can be applied to provide excellent patient care. Specifically, we have attempted to focus on exploring the innovations in the invasive management of aortic regurgitation.

## 1. Introduction

Aortic regurgitation (AR) is also known as aortic insufficiency and results from inadequate closure of the aortic valve leaflets. This promotes volume overload in the left ventricle, leading to subsequent deleterious effects. AR was first described by English surgeon and anatomist William Cowper in 1705 [1]. Meanwhile, the underlying mechanism stems from pathology at the aortic valve leaflets or the aortic root, meaning that as regurgitant blood flows retrograde from the aortic valve back to the left ventricle (LV) in diastole, the LV undergoes remodeling, eccentric hypertrophy, and dilatation. Often, this compensatory mechanism eventually reaches a limit, resulting in a decreased LV systolic function with a reduced LV ejection fraction (LVEF). AR can further manifest as symptoms such as dyspnea, angina, palpitations, presyncope, or syncope. Moreover, AR can progress into advanced diseases, such as elevated cardiac chamber pressures, myocardial ischemia, and decreased cardiac output. This review will explore the etiologies of AR and how imaging modalities can be utilized in diagnosis, risk stratification, surveillance, and intervention for patients with AR. This review also aims to analyze the indications for medical and surgical management and the postoperative surveillance of prosthetic valves.

## 2. Etiologies

The etiologies of AR encompass a wide range of pathological processes affecting the aortic valve leaflets, the aortic root, or both. These etiologies can be classified as valvular causes, aortic root causes, or combined etiologies as the primary drivers of pathophysiology. Thus, understanding the underlying cause of AR is critical for tailoring diagnostic and therapeutic strategies. Each major etiology of AR will be discussed in detail below and are summarized in Table 1.

**Table 1.** **Causes of aortic regurgitation**.

Aortic valve leaflet causes	Aortic root causes
Bicuspid aortic valve	Aortic root dilatation
Endocarditis	Aortic dissection
Rheumatic fever	Aortitis
Myxomatous degeneration	Connective tissue disease
Trauma	Trauma
Calcified aortic valve	Hypertension
Inflammatory/autoimmune	
Prosthetic valve dysfunction	

### 2.1 Valvular Aortic Regurgitation

#### 2.1.1 Congenital (Bicuspid)

The most common congenital defect associated with AR is a bicuspid aortic valve (BAV) [2]. Compared to tricuspid aortic valves, BAVs induce turbulent blood flow patterns, causing higher levels of hemodynamic stress in the valves and the supporting structures [3]. Over time, the elevated hemodynamic stress predisposes the valve to fibrosis and calcification, eventually causing valve dysfunction. Indeed, patients with BAVs are at elevated risk of both aortic stenosis (AS) and AR, often at much younger ages.

In patients with BAVs and unequal leaflet sizes, one leaflet often has a midline raphe resulting from incomplete commissural separation during embryonic development. The orientations of the leaflets vary significantly among patients, with the most common BAV subtype involving fusion of the right and left (R–L) coronary leaflets (59% of cases) and the second most common subtype involving fusion of the right and noncoronary (R–N) leaflets (37% of cases) [4]. The BAV subtype is clinically significant, as the R–N fusion progresses faster than the R–L fusion in terms of both AS and AR, particularly in younger patients [5]. Approximately 32–43% of adults with BAVs develop significant AR, often accompanied by aortic root dilation, which increases the regurgitation volume and worsens the AR [6].

Additionally, the genetic basis of BAV is becoming increasingly recognized. BAVs are usually inherited in an autosomal dominant pattern but with incomplete penetrance and variable expressivity. Meanwhile, mutations in *NOTCH1* and at least 25 other pathways have been implicated in the pathogenesis of BAVs [7, 8]. The systemic relevance of BAVs extends beyond the direct effects, whereby BAVs are also associated with syndromic conditions, such as Turner syndrome. The natural history of BAVs is highly variable, necessitating regular imaging surveillance to identify valvular or aortic disease progression.

#### 2.1.2 Infective Endocarditis

Infective endocarditis (IE) is a critical cause of acute, subacute, and chronic AR. Bacterial and fungal pathogens adhere to the endocardium and valvular apparatus, creating an initial nidus [9]. Once the nidus is established, inflammatory cells and multiple cytokines initiate and mediate an inflammatory response with associated integrins, tissue factors, and adhesion molecules. The inflammatory response propagates, attracting monocytes and platelets, which leads to thrombus formation, creating a favorable environment for infectious pathogens to reside in, and finally, forming infected vegetation on the surface of the valve [10].

This vegetation can erode or perforate the leaflets, resulting in acute, severe AR. Occasionally, leaflet rupturing can occur, precipitating an abrupt hemodynamic collapse due to acute left ventricular volume overload [11]. In these severe cases, IE causes low cardiac output, a significant rise in left ventricular end-diastolic pressure (LVEDP), and cardiogenic shock [12]. The clinical markers that highlight the level of severity of AR include increasing heart rate (≥94 bpm) and diastolic mitral regurgitation (DMR), which should gauge decision outcomes for emergent surgical intervention [12]. Conversely, chronic cases may stem from scarring and deformity following infection resolution.

Prompt diagnosis through echocardiography and blood cultures is essential, as untreated IE carries a high mortality rate. Research has shown that persons who inject drugs (PWID) present an elevated risk of recurrent IE, while fungal IE is more prevalent in second-episode endocarditis and associated with increased mortality [13]. *Candida albicans* is a common mycological pathogen that frequently contaminates illicit opioid drugs and, therefore, is the culprit of most fungal IE and potential valvular vegetations with fungal origins [13].

#### 2.1.3 Rheumatic Fever

Rheumatic heart disease (RHD) due to rheumatic fever (RF) is most often caused by *Streptococcus pyogenes* (group A strep, GAS). However, substantial scientific evidence supports a rise in RHD cases involving group C and even group G streptococci [14]. Molecular mimicry has long been the accepted immunological pathway of RHD. Meanwhile, new evidence highlights the linkage between the streptococcal M-protein type 3 (M3-protein) and its interaction with the CB3-region in collagen type IV (CIV) [15]. Rheumatic fever can result in fibrotic changes to the aortic valve, which is usually a chronic phenomenon, leading to the classic finding of fusion of the aortic valve commissures. The fibrosed aortic valve cusps prevent proper forward cardiac flow, representing aortic stenosis associated with rheumatic fever. Likewise, these fibrosed aortic valve cusps prevent proper valve closure during diastole, which manifests as chronic aortic regurgitation. Mortality due to RHD presents a heavy burden, especially on low-income countries, with an annual reported mortality rate of 300,000 patients per year [16].

#### 2.1.4 Myxomatous and Degenerative

Myxomatous valve degeneration occurs when a native valve becomes fibrosed by depositing extracellular matrix components, such as glycosaminoglycans, and loses its intrinsic structural integrity. Recent research has shown a link between certain transcription factors and the risk of developing cardiac valve myxomatous degeneration [17]. If this valvular degeneration develops in the aortic valve, it can predispose patients to developing AR. In rare cases, cardiac myxomas can also contribute to AR; thus, prompt surgical intervention is warranted [18]. In the pediatric population, myxomatous degeneration of cardiac valves has been linked to genetic diseases such as trisomy 18, Noonan, Marfan, and Ehlers–Danlos syndrome, with a huge emphasis on the 6q25.1 gene deletion that expresses the transforming growth factor (TGF)-beta-activated kinase 1/mitogen-activated protein kinase kinase kinase 7 (MAP3K7)-binding protein 2 (*TAB2* gene) protein [19].

#### 2.1.5 Traumatic

AR, resulting from trauma, occurs due to direct damage to the valve apparatus, including cusp avulsion or annular disruption. Multiple case reports have described patients suffering from high-impact force attributed to motor vehicle collisions, mostly sustaining crushing chest injuries that have resulted in AR and eventual deterioration into cardiogenic shock [20, 21, 22]. These patients usually require emergent surgical intervention.

### 2.2 Aortic Root Disease

#### 2.2.1 Aortic Dissection

Thoracic aortic dissection (AD) is a critical cause of acute AR and should be considered in all patients presenting to the emergency department with acute chest or back pain. AD occurs when a tear in the intimal layer of the aortic wall allows blood to enter and “dissect” between the media layers, creating a false lumen. This disruption frequently extends to the aortic root, annulus, or commissures, leading to leaflet malcoaptation and acute, severe AR [23]. Clinical presentation often includes severe chest pain, described as a tearing or ripping sensation, which radiates to the back. Diagnosis is typically made using contrast-enhanced computed tomography (CT) angiography, which provides high-resolution images of the aorta. Transesophageal echocardiography (TEE) is another valuable tool, particularly in unstable patients, as it can assess both the aortic valve and dissection flap in real time [24]. Prompt diagnosis and localization of the dissection are crucial, since location assists with the classification of the dissection and guides management [25].

#### 2.2.2 Cystic Medial Necrosis

Cystic medial necrosis is a degenerative condition characterized by the fragmentation of elastic fibers and the accumulation of the mucoid extracellular matrix in the medial layer of the aortic wall. These changes weaken the structural integrity of the aorta, predisposing it to dilation, aneurysm formation, and dissection [26]. The pathophysiology of cystic medial necrosis is linked to genetic mutations, particularly in fibrillin-1 (FBN1) in Marfan syndrome and TGF-β signaling pathway mutations in Loeys–Dietz syndrome. These mutations disrupt the production or function of extracellular matrix proteins, contributing to the loss of medial integrity. Characteristically, the diseased and weakened portion of the aorta occurs at the level of the aortic root and proximal ascending aorta. Over time, the aortic root and ascending aorta are remodeled under pulsatile aortic blood flow, creating an aneurysm. The wide dilatation of the aortic root and, subsequently, the aortic valve annulus causes AR [27].

#### 2.2.3 Osteogenesis Imperfecta

Osteogenesis imperfecta (OI) is a genetic disorder resulting from gene mutations encoding type 1 collagen, COL1A1, and/or COL1A2. While OI primarily affects the skeleton, it also has significant cardiovascular implications due to the role of type I collagen in maintaining the structural integrity of the vascular wall, aortic root, and valve annulus; the aortic root is particularly vulnerable in OI [28]. Meanwhile, numerous studies have shown increased aortic root diameters in patients with OI, resulting in progressive dilation and AR over time [28, 29]. The severity of cardiovascular involvement varies with the type of OI, with moderate-to-severe forms (types III and IV) more commonly associated with aortic complications [29].

#### 2.2.4 Severe Chronic Hypertension

Long-standing, untreated hypertension may play a role in progressive aortic root dilatation [30, 31]. This may be due to continuous pressure overload, leading to accelerated vascular stiffening and loss of elastic fibers in the vasculature. Furthermore, endothelial dysfunction is a well-known risk factor for hypertension, and this may play a role in the pathogenic remodeling process. Collectively, these processes may result in greater annular stretching and degenerative valve changes, leading to the development and progression of AR [32].

#### 2.2.5 Syphilis

Syphilitic aortitis represents a severe cardiovascular manifestation of tertiary syphilis, arising from chronic inflammation of the vasa vasorum. The small vessels that supply the outer layers of the aortic wall become inflamed, leading to obliterative endarteritis. This results in ischemic injury, medial necrosis, and fibrotic scarring, ultimately weakening the structural integrity of the ascending aorta [33]. Over time, these processes cause aortic root dilation and proper aortic valve leaflet coaptation failure, leading to progressive AR [34]. Syphilitic AR is often associated with ascending aortic aneurysms, which increase the complexity of management and contribute to significant morbidity.

Historically, syphilis was one of the leading causes of AR and valvular disease before the advent of antibiotics. The widespread use of penicillin and improved screening methods in the mid-20th century led to a dramatic decline in syphilis-related cardiovascular complications in developed countries. However, syphilitic aortitis and AR remain important concerns, particularly in resource-limited settings. Collectively, tertiary syphilis still occurs in 10–30% of untreated syphilis patients [35]. Among patients with tertiary syphilis, approximately 10–15% develop cardiovascular manifestations.

### 2.3 Other Causes

#### 2.3.1 Functional LV Dilation (Secondary AR)

Functional AR results from left ventricular dilation that disrupts the normal mechanics of the aortic valve without primary valve disease. This condition is often seen in severe LV dysfunction cases caused by dilated cardiomyopathy, myocarditis, or ischemic heart disease [36]. In functional AR, the dilated LV pulls the aortic annulus apart, preventing complete coaptation of the valve leaflets during diastole. Additionally, aortic annular dilation is compounded by increased wall stress and remodeling, further exacerbating regurgitation [36]. Functional AR typically develops gradually and reflects the underlying disease progression in LV dilation. Symptoms are often overshadowed by those of heart failure, including exertional dyspnea, fatigue, and orthopnea. The regurgitant volume is frequently proportional to the degree of annular and ventricular enlargement; meanwhile, diagnosis relies on echocardiographic findings, which normally comprise LV dilatation, dilated cardiomyopathy, and aortic annulus dilatation [37].

#### 2.3.2 Radiation Induction

Patients undergoing radiation therapy are at elevated risk of radiation-induced AR. Radiation damages the connective tissue and microvasculature of the aortic valve and ascending aorta, leading to fibrosis, scarring, and calcification. Over time, these changes can distort the valve anatomy, impair leaflet mobility, and cause AR [38]. Patients with radiation-induced AR often present decades after completing radiation therapy, with symptoms that mirror those of chronic AR. Therefore, diagnosis requires a high index of suspicion in patients with a history of mediastinal radiation. Notably, radiation damage often extends to surrounding cardiac structures, complicating the clinical presentation [39].

#### 2.3.3 Ankylosing Spondylitis

Ankylosing spondylitis, a chronic inflammatory disease primarily affecting the axial skeleton, can also involve the cardiovascular system and promote AR in some patients. Inflammatory changes in ankylosing spondylitis typically affect the aortic root and ascending aorta, leading to annular dilation, aortic wall thickening, and aortic valve leaflet fibrosis. These changes impair leaflet coaptation, resulting in progressive AR. Studies suggest that approximately 2–10% of patients with ankylosing spondylitis develop significant AR, with a higher prevalence observed in patients with long-standing or severe disease [40]. Imaging studies, including echocardiography, are essential for evaluating AR severity, while MRI may be used to assess spinal or joint involvement [41].

## 3. Clinical Presentation

The clinical presentation of AR can vary depending on the chronicity of the disease and the disease severity. Acute AR is a critical medical condition that demands prompt identification and intervention to avert rapid deterioration, including death. Comparatively, chronic AR can remain asymptomatic for decades as the LV adapts to the regurgitant volume before these compensatory mechanisms fail. The presentations of both chronic and acute AR are discussed below.

### 3.1 Acute Aortic Regurgitation

Acute AR, most commonly caused by infectious endocarditis, aortic dissection, or aortic valve damage from trauma, usually results in a medical emergency. The sudden structural or functional impairment of the aortic valve leads to rapid diastolic backflow into the LV, which prevents compensatory mechanisms from developing. Hemodynamically, this leads to rapid decompensation through several mechanisms. Firstly, left ventricular volume overload occurs rapidly during acute AR. During diastole, the LV receives blood from both the left atrium and regurgitant flow from the aorta, resulting in acute volume overload. The sudden increase in end-diastolic volume causes an immediate rise in LVEDP, which is transmitted retrogradely to the pulmonary circulation, leading to pulmonary congestion and edema. As the volume rapidly increases, so does the left ventricular pressure and wall stress. This abrupt increase in LV wall tension due to the elevated volume and pressure exacerbates myocardial oxygen demand while impairing coronary perfusion during diastole, which can lead to myocardial ischemia and reduced contractility. Finally, acute AR can result in compromised mitral valve function. The elevated LV pressure during diastole may cause premature mitral valve closure, altering its normal function and leading to a soft first heart sound (S1).

#### Physical Examination Signs in Acute AR

Physical examination findings in acute AR patients are distinct and reflect the hemodynamic urgency of the condition. The pertinent physical examination findings of acute AR are detailed in Table 2.

**Table 2.** **Physical examination findings of acute aortic regurgitation**.

Category	Findings	Description
General appearance	Appear critically ill, with distress, diaphoresis, and cyanosis	Indicative of systemic hypoperfusion and respiratory compromise
Cardiac auscultation	Diastolic murmur	Short, high-pitched, decrescendo murmur best heard at the left sternal border during early diastole
	Soft S1 sound	Premature mitral valve closure dampens the intensity of the first heart sound
	Absent or faint S3/S4	Occurs due to limited time for ventricular adaptation
Pulse characteristics	Absence of bounding pulses and narrow pulse pressure	Reduced stroke volume, and diastolic pressure remains elevated
Pulmonary findings	Rales or crackles	Bilateral crackles indicative of pulmonary edema, extending to apices in severe cases
	Tachypnea and respiratory distress	Reflective of pulmonary congestion and hypoxemia
Signs of hypoperfusion	Cool, clammy extremities with delayed capillary refill	Signals poor systemic perfusion secondary to low cardiac output
Systemic signs	Hypotension and tachycardia	Result of compensatory sympathetic activation
	Cyanosis and altered mental status	Develops in advanced cases as cardiac output declines

S1, first heart sound.

### 3.2 Chronic Aortic Regurgitation

Chronic AR develops gradually, allowing time for the LV to employ compensatory mechanisms to accommodate the increased volume load caused by the regurgitant flow. The primary adaptive response involves eccentric hypertrophy, characterized by cardiomyocyte elongation and thinning, leading to LV dilatation. This structural remodeling enables the ventricle to maintain stroke volume and cardiac output despite the increased regurgitant volume. Notably, patients often remain asymptomatic during this compensatory phase, sometimes for as long as 10–15 years. However, these compensatory mechanisms are not indefinite, and the persistent volume overload over time results in progressive myocardial dysfunction, marked by rising LVEDP and declining LVEF. Subsequently, symptoms emerge when this transition to decompensation occurs. Patients typically report exertional dyspnea due to elevated pulmonary pressures, fatigue from reduced forward cardiac output, and orthopnea or paroxysmal nocturnal dyspnea associated with pulmonary congestion. Palpitations may also be reported, driven by an augmented stroke volume or arrhythmias, such as premature ventricular contractions, which are common in advanced disease states.

#### Physical Examination Signs in Chronic AR

Findings from physical examination of patients with chronic AR reflect the hyperdynamic circulation caused by the regurgitant flow and the compensatory changes in the LV. These findings are summarized in Table 3.

**Table 3.** **Physical examination findings of chronic aortic regurgitation**.

Category	Findings	Description
Pulse characteristics	Corrigan’s pulse (water hammer pulse)	Bounding, rapid upstroke, and collapse in the arterial pulse due to wide pulse pressure
	De Musset’s sign	Rhythmic head bobbing in synchronization with the heartbeat caused by exaggerated arterial pulsations
	Quincke’s sign	Visible capillary pulsations in the nail beds when pressure is applied to the distal nail plate
	Muller’s sign	Pulsation in the uvula observed during cardiac systole
Cardiac auscultation	Diastolic murmur	High-pitched, blowing decrescendo murmur best heard at the left sternal border
	Austin Flint murmur	Low-pitched, mid-to-late diastolic rumble at the apex caused by the regurgitant jet
	Systolic murmur	Ejection murmur due to increased forward flow across the aortic valve
Physical examination	Wide pulse pressure	Increased systolic and decreased diastolic pressures from the augmented stroke volume
	Displaced apical impulse	Forceful and sustained impulse due to left ventricular dilation
	Lateral and inferior apical displacement	An enlarged left ventricle displaces the point of maximal impulse laterally and inferiorly

## 4. Classification of Chronic AR (as per Current AHA/ACC Guidelines, 2020)

Classifying chronic AR is essential for delineating disease progression, predicting long-term clinical outcomes, and tailoring management. Current guidelines stratify chronic AR into four progressive stages (A through D), each reflecting specific clinical and hemodynamic features. Stages A–D are summarized below in reference to the 2020 American College of Cardiology (ACC) and American Heart Association (AHA) Guidelines for the management of patients with valvular heart disease [42].

Stage A, termed “at risk”, encompasses individuals with structural valve abnormalities but no evidence of regurgitation or clinical sequelae of the disease. At this stage, these patients have no hemodynamic consequences. “At-risk” members in this stage include patients with congenital bicuspid aortic valve, mild aortopathy, early rheumatic changes, and diseases of the aortic sinuses or ascending aorta. Surveillance focuses on identifying patients transitioning to more advanced stages [42].

Stage B refers to asymptomatic “progressive AR”. Stage B is characterized by mild to moderate regurgitation, preserved LVEF, and normal or mildly dilated LV dimensions. Symptoms are absent, but Doppler echocardiography often reveals mild diastolic flow reversal in the descending aorta and regurgitant jets with specific characteristics. In stage B patients with mild AR, the left ventricular outflow tract (LVOT) jet width is <25%, and the regurgitant volume is <30 mL/beat. In stage B patients with moderate AR, the LVOT jet width is 25%–64%, and the regurgitant volume is 30–59 mL/beat. Patients in stage B with moderate AR require periodic monitoring to detect disease progression [42].

Stage C denotes asymptomatic severe AR and is subdivided into C1 and C2. C1 includes patients with normal LVEF (>55%) and mild to moderate LV dilation characterized by a LV end-systolic diameter (LVESD) of <50 mm; these patients are often identified incidentally during echocardiographic evaluation. C2 includes patients with abnormal LV systolic function with depressed LVEF (≤55%) or severe LV dilation (LVESD >50 mm or indexed LVESD >25 mm/m^2^). Moreover, stage C2 indicates advanced myocardial remodeling and reduced compensatory capacity, while subclinical symptoms may emerge on stress testing [42].

Stage D, or “symptomatic severe AR”, represents the most advanced stage, with patients reporting dyspnea, angina, fatigue, or heart failure symptoms. Echocardiographic findings include severe regurgitant volume (≥60 mL/beat), regurgitant fraction (≥50%), and diastolic flow reversal extending to the abdominal aorta. Significant LV dilation, wall thinning, and reduced LVEF are also typical. This stage demands immediate evaluation for surgical intervention [42].

Advances in imaging modalities, including cardiac magnetic resonance imaging and three-dimensional echocardiography, have enhanced the ability to stratify AR severity accurately and assess the interplay between valvular dysfunction and ventricular remodeling. These classifications provide a structured framework to ensure timely intervention, particularly as patients progress to stages C2 and D. At these stages, any delay in therapy can result in significant morbidity and mortality [42].

## 5. Diagnosis

According to the 2020 ACC/AHA guidelines for managing valvular heart disease, transthoracic echocardiography (TTE) is indicated in patients with signs/symptoms of AR. In moderate to severe AR with equivocal TTE findings, these patients warrant further workup using TEE, cardiac magnetic resonance imaging, or angiography [42].

### 5.1 Transthoracic Echocardiography

**Fig. 1.**
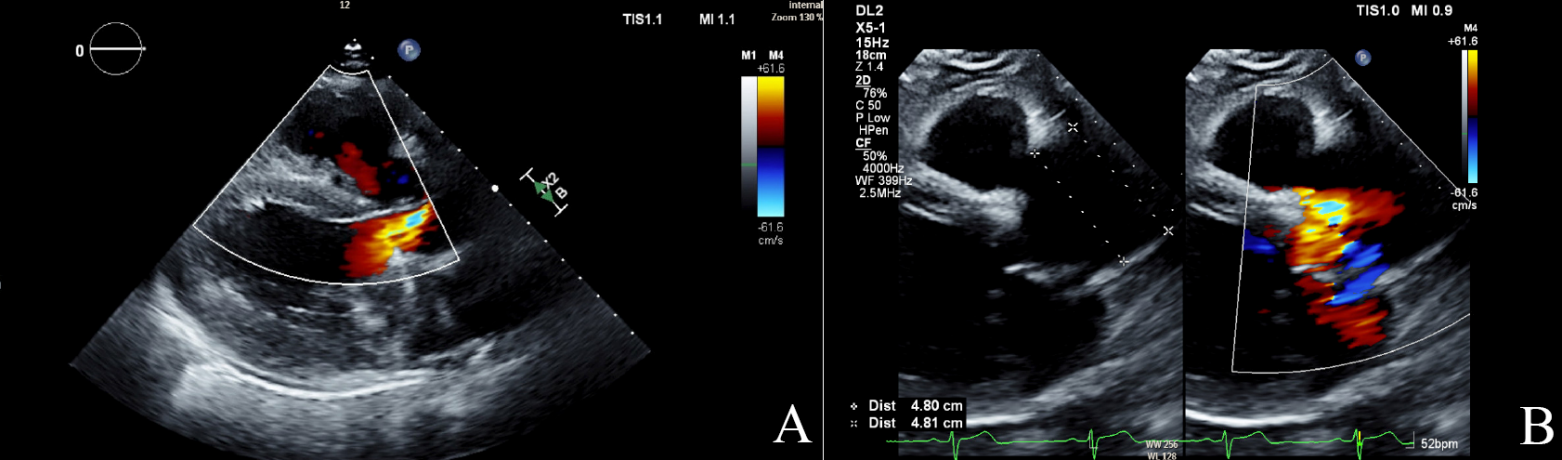
**Echocardiographic findings in aortic regurgitation**. (A) Color Doppler image of aortic regurgitation jet (mosaic color) in parasternal long axis (PSLAX). (B) The PSLAX view shows a dilated aortic root measuring 4.8 cm with a regurgitant jet in mosaic. TIS, thermal index soft tissue; MI, mechanical index; DL2, display layout 2; 2D, two-dimensional; CF, color flow; WF, wall filter.

TTE forms the first-line diagnostic modality indicated for valvular disorders such as AR. TTE can also be used to determine the presence, severity, and cause of AR [43]. Fig. 1 shows the echocardiogram findings of AR in a patient with aortic leaflet dysfunction and dilated aortic root. Color flow Doppler imaging can identify the presence of a regurgitant jet at the aortic valve during diastole and measure the jet width, jet area, and vena contracta (VC) width [43]. These measurements tend to be reliable with a central jet. However, the AR severity could be underestimated if there is an eccentric jet, which is a regurgitant AR jet traveling along the posterior aspect of the LVOT or the anterior leaflet of the mitral valve [44]. If there is evidence of an eccentric jet, a measurement of the proximal isovelocity surface area (PISA) can be used to assess the AR severity. Continuous wave Doppler can be used to measure pressure half-time (PHT), deceleration time, and signal density [43, 45]. Pulsed wave Doppler via the suprasternal view is useful in determining the degree of holodiastolic flow reversal in the descending thoracic compared to the forward systolic flow, as well as the regurgitant volume, regurgitant fraction, and effective regurgitant orifice area (EROA) [43]. The M-mode can be used to evaluate for fluttering, a component of the Austin Flint murmur, and premature mitral valve closure caused by the regurgitant AR jet [43].

TTE is useful for determining the LV size and systolic function, aortic root dilatation, aortic dissection, endocarditis, and other leaflet pathologies [42]. In chronic AR, the LV systolic function is initially normal or even slightly above normal. However, remodeling occurs over time to compensate for the increased LV pressure and volume. Thus, the LV size increases, and LVEF decreases. In contrast, the TTE findings in acute AR demonstrate significant regurgitation but normal LV size and systolic function [46]. A shorter PHT indicates rapid equilibration of the aortic and LV diastolic pressures, thus corresponding to more severe AR. This measurement is especially useful in acute AR; in chronic AR, the LV has undergone prolonged remodeling, which allows it to adjust for the increased diastolic pressures [47]. Certain measures for AR, including regurgitant volume (RV) and EROA, are predominantly used in the workup of chronic AR, as these measurements can be unreliable in acute AR [46]. Semiquantitative and quantitative measures can be utilized to stratify the severity of AR, as seen in Table 4 (Ref. [48]).

**Table 4.** **Semiquantitative and quantitative parameters for aortic regurgitation severity [48]**.

Parameters		Mild	Moderate	Severe
Semiquantitative				
	Vena contracta width (mm)	<3 mm	3–6 mm	>6 mm
	Pressure half-time (ms)	>500 ms	500–200 ms	<200 ms
Quantitative				
	Effective regurgitant orifice area (mm^2^)	<10 mm^2^	10–30 mm^2^	>30 mm^2^
	Regurgitant volume (mL)	<30 mL	30–59 mL	≥60 mL

Echocardiographic findings are also used to predict outcomes, in addition to utilizing TTE to determine the prevalence and severity of AR. Indeed, the LVEF has been well-documented as a long-term predictor of survival in patients with chronic AR [49, 50]. In asymptomatic patients, quantitative American Society of Echocardiography (ASE) measurements, such as RV and EROA, were used to show a positive correlation between AR severity and an increased risk of mortality and cardiac events [51]. Other echocardiographic measures, including LV end-systolic volume index (LVESVi) and linear LV end-systolic dimension index (LVESDi), are associated with increased mortality risk [52, 53].

Chronic AR can progress over many years with new or worsening symptoms, increased severity, reduced LVEF, and LV enlargement [54]. This progression over time necessitates serial echocardiographic surveillance of AR, as the appropriateness for surgical intervention can change as symptoms, severity, and LV size and function worsen [42, 55, 56]. Moreover, the American and European guidelines vary slightly regarding the screening frequencies. Current recommendations for AR screening (for patients in which surgery is not yet indicated) can range anywhere from every 3–6 months to every 3–5 years, depending on AR severity and/or significant worsening of LV dysfunction or size [42, 57]. In patients meeting the criteria for surgical intervention, it is imperative to integrate shared decision-making in a patient-centered approach.

While TTE remains the gold standard in diagnosing AR, it has limitations. Indeed, poor acoustic windows or suboptimal image quality with TTE may necessitate the utilization of another imaging modality. Additionally, volumetric measurements obtained using TTE may display some variability in operator dependence and reproducibility compared with other modalities [58].

### 5.2 Transesophageal Echocardiography

TEE is an alternative diagnostic tool in AR evaluation. If TTE is inconclusive or has poor acoustic windows, TEE can be utilized to obtain quantitative measures for identifying the presence and severity of AR [42, 59]. TEE can also further identify the aortic leaflet or root pathologies [43]. The diagnostic utility of TEE in evaluating endocarditis has long been established, and presents high sensitivity and specificity in identifying valvular vegetations [60, 61, 62]. In addition, TEE is useful in preoperatively evaluating the aortic valve and root structural anatomy for anticipated surgical interventions [43, 46]. Specifically, TEE is used in the presurgical evaluation to establish the underlying mechanism of AR, guide the operative approach in valve repair or replacement, and predict postoperative outcomes [63]. TEE also represents a valuable tool in assessing for dysfunction or misalignment of a prosthetic valve [46].

### 5.3 Computed Tomography

The information extracted from imaging studies, such as CT, is beneficial in precisely mapping the aortic size and morphological features of the AR whilst assessing the risk of coronary artery disease [64]. Maximum diameter measurements of the aortic valve are crucial for surgical planning and are divided into four levels: annulus, sinus of Valsalva, sino–tubular junction, and tubular ascending aorta [64]. CT imaging can also shed light on the pathological state of the aortic valve, such as calcifications, prolapse, infectious etiology, and/or rheumatic disease, with an emphasis on utilizing retrospective electrocardiographic (ECG)-gated CT as the best choice to pinpoint such findings rather than a higher radiation dose [64].

### 5.4 Cardiac Magnetic Resonance Imaging (CMR)

CMR imaging can be used as a diagnostic tool in AR when there is inadequate data from other imaging modalities. This includes situations with suboptimal echocardiographic images, poor acoustic windows, or discordance between echocardiographic and clinical findings. CMR imaging has also been validated as a reliable modality for evaluating valvular disorders such as stenosis and regurgitation [65]. Indeed, CMR imaging can be used for AR patients to assess aortic valvular or root pathology, AR severity, LV size, function, and remodeling [43, 65]. CMR imaging can also be used to evaluate for holodiastolic flow reversal in the descending aorta, which strongly predicts severe AR and mortality [66, 67].

Notably, CMR has consistently produced reliable and reproducible results in multiple studies comparing the reliability of imaging findings to the well-established modality of Doppler echocardiography [68, 69, 70, 71, 72]. Moreover, compared to TTE, CMR imaging has demonstrated less variability in assessing regurgitant volume measurements [58, 73]. Additionally, CMR assessment of the regurgitant volume is more reliable than TTE, which may be due to the ability of CMR imaging to better localize the imaging slice to the correct aortic level [74, 75, 76, 77].

Long-term LV volume overload due to chronic AR can cause LV remodeling, which is mediated by myocardial fibrosis and predisposes to the development of heart failure [78, 79, 80]. Thus, investigating the extent of LV remodeling with CMR is important in evaluating AR [43, 81]. The degree of LV remodeling can be quantified using CMR imaging to determine the LVESVi. In asymptomatic or minimally symptomatic patients, the CMR measurement of the LVESVi and regurgitant fraction was associated with symptom progression and all-cause mortality [82]. Indeed, one study demonstrated that CMR imaging was superior to TTE in evaluating LV mass and remodeling [83].

Similar to serial echocardiography, CMR imaging can quantify the degree of chronic AR progression and determine the timing of surgical intervention. Moreover, CMR imaging can reliably predict outcomes and surgical requirements [84, 85]. In fact, CMR imaging can also be utilized post-transcatheter aortic valve implantation (TAVI) to assess for AR severity, paravalvular regurgitation, and LV function and size [86, 87].

While CMR imaging is an effective tool in evaluating AR etiology, severity, intervention, and screening, there are limitations to this modality; for example, the cost of CMR imaging is significantly increased compared with TTE, TEE, and CT. Additionally, unlike the previously discussed imaging modalities, CMR imaging is not currently widely available at all facilities. Nonetheless, as the healthcare industry continues to innovate and advance, CMR imaging will likely become a mainstay in the diagnostic approach to AR.

### 5.5 Aortography

Updated societal guidelines suggest the reservations of cardiac catheterization with LV and aortic angiography for situations with inconclusive findings despite extensive diagnostic workup or when there is clinical concern for acute coronary syndrome (ACS) [42, 88]. Aortic angiography, or aortography, can assess hemodynamic measurements, AR severity, aortopathy, and LV size and function [89, 90]. Aortography was once considered a mainstay in the diagnostic workup of AR. However, aortography has since been overtaken by TTE and CMR imaging due to the reliability of these noninvasive imaging modalities. Thus, the modern indication of aortography is mainly for evaluating paravalvular leaks (PVLs), using the video-densitometry technique for monitoring after TAVI [91]. This technique can be utilized to obtain quantitative measurements, which predict post-TAVI outcomes, including mortality [92, 93, 94]. Thus, given these possible outcomes, aortography findings can be used to manage post-TAVI patients and evaluate for any indication of repeat valvular intervention.

## 6. Clinical Approach to the Management of AR

The management of AR follows a comprehensive approach that factors in chronicity, clinical presentation, and objective data: this can include a medical, surgical, or combined approach.

Acute AR: Surgical intervention to treat acute severe AR should not be delayed, especially in patients with red flag signs involving hemodynamic instability, such as hypotension, pulmonary edema, or low flow state [42].

Chronic AR: Management depends on the stage, regurgitant severity, clinical symptoms, and LV size and function [42].

### 6.1 Medical Management

The key mechanism for pharmacologically managing AR is to reduce afterload on the LV, thus promoting forward cardiac output and discouraging regurgitant flow. Current guidelines recommend treating patients with chronic AR stages B and C using an antihypertensive regimen for systolic blood pressure (SBP) above 140 mmHg [42].

The mainstays of treatment include using an angiotensin-converting enzyme inhibitor (ACE-i) or an angiotensin II receptor blocker (ARB). Many studies have shown the benefits of afterload-reducing agents in managing patients with AR and coexisting hypertension. These include decreased mortality, reduced LV volume overload, improved LVEF and cardiac output, decreased degree of regurgitation, and decreased myocardial oxygen demand [95, 96, 97, 98, 99, 100, 101]. Moreover, when used in patients with chronic severe AR, beta-blockers (BBs) improved survival, mainly in patients with higher heart rates [102]. Interestingly, BBs are avoided in acute AR caused by etiologies other than aortic dissection, as these drugs can attenuate the appropriate tachycardic compensatory response [42].

Further long-term studies have shown that the pharmacological approach does not decrease or delay the need for valve intervention in patients with chronic, severe AR [103]. Thus, in addition to medical management, the surgical approach plays a significant role in managing AR.

### 6.2 Surgical Management

#### 6.2.1 Indications for Intervention

**Fig. 2.**
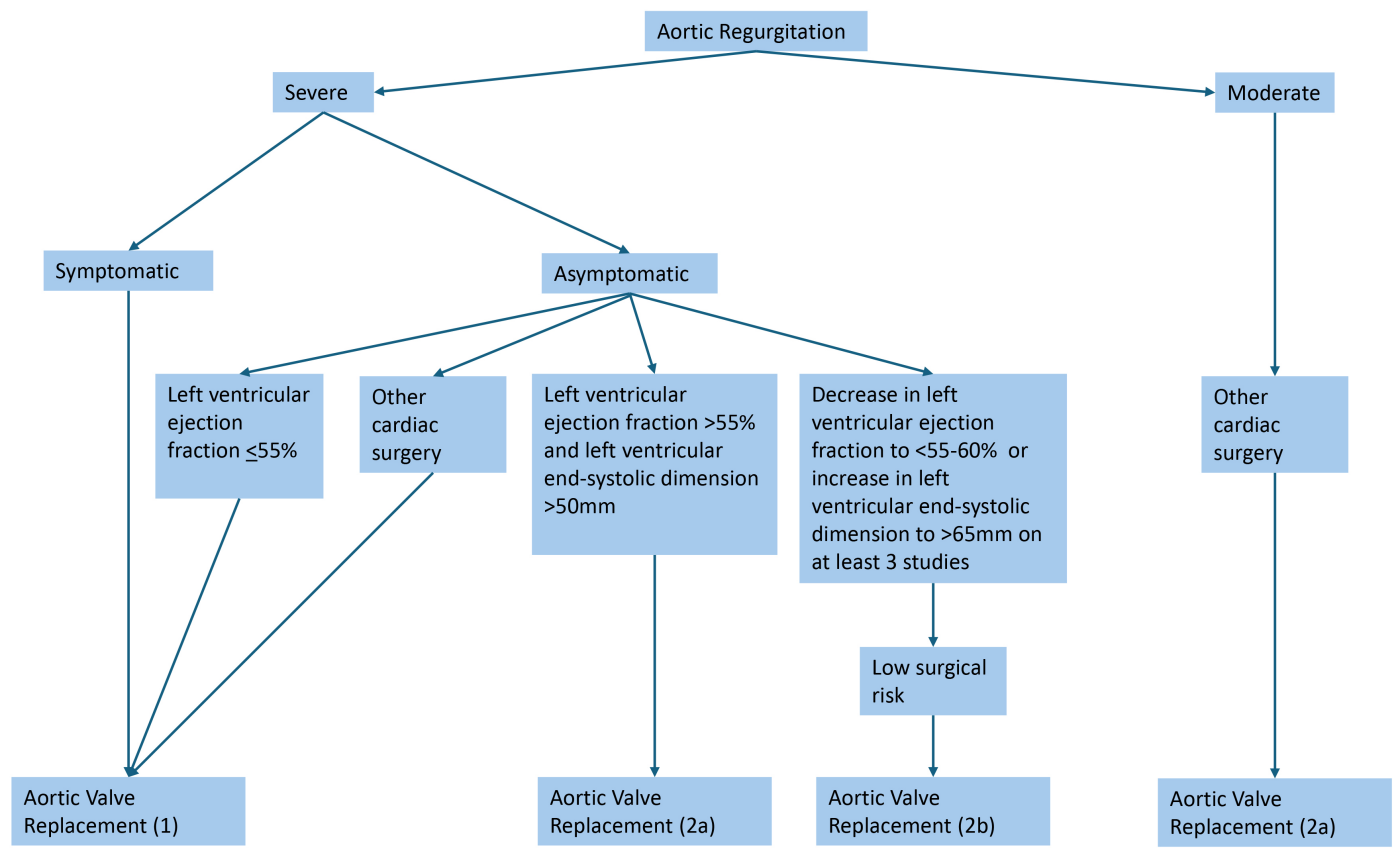
**Indications for aortic valve replacement in aortic regurgitation [42]**. Severe aortic regurgitation is defined as a vena contracta length greater than 0.6 cm, holodiastolic aortic flow reversal, a regurgitant volume greater than or equal to 60 mL, a regurgitant fraction greater than or equal to 50%, and an effective regurgitant orifice area greater than or equal to 0.3 cm^2^.

According to the updated 2020 AHA/ACC guidelines, recommendations for valvular intervention in AR are largely based on severity, symptoms, LV function, and size (Fig. 2; Ref. [42]). Aortic valve replacement (AVR) is indicated in symptomatic severe AR (stage D), asymptomatic severe AR with LV dysfunction or dilation (stage C2), asymptomatic severe AR with normal LV function (stage C1) but with progressively worsening LVEF or dilation, and moderate AR that is also undergoing surgery for other concurrent cardiac issues [42]. Shared decision-making with patients, considering patient-specific factors such as age, surgical risk, and risk factors, is an important aspect of preoperative evaluation.

#### 6.2.2 Considerations for Earlier Intervention 

While societal guidelines recommend valvular intervention for AR based largely on symptoms and echocardiographic findings, there is a growing belief amongst clinicians that interventions should be pursued even before deleterious LV remodeling occurs. One study that evaluated young patients with chronic AR who underwent AVR showed that patients with higher baseline LVESD values were less likely to achieve improvement after valve replacement [104]. Another study demonstrated that, among patients who underwent surgical aortic valve replacement (SAVR), those who met either class II or no indication had better survival outcomes than those who met the class I indication for AVR [105].

These findings suggest that pursuing an earlier intervention may be beneficial in preventing the development of clinical symptoms, heart failure, and death. This may be partly due to the formulation of the current guidelines using older studies, which do not consider the contemporary advances in invasive management. While the 2020 AHA/ACC guidelines did progress in updating parameters from previous guidelines, future societal guidelines should aim to advance these recommendations further to favor earlier intervention.

#### 6.2.3 Innovations in Transcatheter Heart Valves

Significant recent advancements have been made in developing transcatheter heart valves (THVs) used for transcatheter aortic valve replacement (TAVR). Previously, standard bioprosthetic valves were used for TAVR to treat both AS and AR; however, structural differences between AS and AR limit the reliability of the same valve in both pathologies. For example, TAVR valves in AS rely on the calcification of the aortic valve to allow for the replacement valve to be seated properly. In contrast, AR (in the absence of concomitant AS) does not have significant valvular calcification; thus, there can be challenges with ensuring stabilization of the replacement valve. For these reasons, innovation in TAVR valve technology for AR has been an area of growing research and industry interest.

The JenaValve Trilogy system is a novel, dedicated THV developed to treat pure AR [106]. Once deployed, the device clips onto the native aortic valve leaflets to ensure anchoring within the annulus; early results show favorable outcomes for technical success and survival [106, 107, 108, 109].

Meanwhile, the J-Valve system represents another recently introduced THV for pure, native AR [110]. This device is a self-expanding porcine valve with anchor rings to assist with proper seating within the annulus. Studies have shown high procedural success rates and survival, and a low rate of complications [111, 112, 113].

In treating native AR, dedicated THV outperforms off-label TAVR valves in outcomes including procedural success, reintervention rates, and mortality [114, 115]. While these novel THVs offer an exciting innovation to the invasive AVR approaches, further studies are needed to assess the long-term outcomes of these valves. Large-scale studies are ongoing to evaluate these devices further using real-world data.

#### 6.2.4 TAVR vs. SAVR

The catheterization and surgical approaches used in AR interventions are associated with TAVR and SAVR, respectively. TAVR involves inserting a bioprosthetic aortic valve replacement, whereas SAVR involves inserting either a bioprosthetic or mechanical aortic valve.

TAVR is generally recommended in patients older than 80 years of age with an expected life expectancy of less than 10 years, as older patients have a high risk of morbidity and mortality with SAVR [42, 116]. Conversely, the current guidelines highlight the importance of SAVR as the preferred treatment method in patients under 65 years old with a life expectancy greater than 20 years.

The use of TAVR to treat AR can pose unique challenges. Some repercussions include the formation of acute transcatheter valve embolization or migration (TVEM) [117]. Additionally, aortic dilatation and possible valve malpositioning pose significant risks of causing paravalvular regurgitation or leaks. These complications can be a major cause of morbidity and mortality after aortic valve replacement, and regular post-procedural monitoring is often indicated to screen for complications.

#### 6.2.5 Post-Surgical Surveillance

Postoperative evaluation of prosthetic aortic valves via either SAVR or TAVR is primarily performed using Doppler echocardiography. Initially, a TTE is performed within 6–12 weeks after patients undergo aortic valve replacement, which serves as a new baseline against which future studies can be compared. Echocardiographic information that is obtained for the evaluation of the prosthetic valve includes LV systolic function and size, pressure gradients, valvular velocities, jet contour, regurgitant severity, opening and closing of valve leaflets, leaflet thickening, valve obstruction, paravalvular regurgitation, and prosthetic valve position [118]. TEE should also be considered if the patient develops heart failure symptoms or if TTE reveals abnormal transvalvular gradients.

The subsequent echocardiographic surveillance intervals are performed using a patient-specific, dynamic approach.

For the bioprosthetic SAVR valves, it is generally recommended to repeat TTE 5 years after the initial study, and then again after 10 years, before continuing surveillance annually [42]; annual TTE is recommended for bioprosthetic TAVR valves [42]. Comparatively, regular surveillance is not indicated for mechanical prosthetic valves, and general recommendations, as seen in the other valve types, suggest obtaining repeat imaging if new signs or symptoms concerning valvular or myocardial dysfunction develop. As discussed, alternative imaging modalities, including TEE, CT, or CMR, may be utilized for suboptimal TTE findings.

## 7. Conclusion

AR is a valvular disorder that can progress over time, eventually causing LV dilatation and dysfunction. AR can result from acute or chronic pathologies of the aortic leaflets or the aortic root. Thus, accurate diagnosis and severity grading are warranted in the workup of AR to deliver the appropriate intervention.

TTE represents the first-line tool for diagnosis, severity, and monitoring; however, other imaging modalities can be utilized if inadequate TTE findings are present. Further, TEE is a strong diagnostic tool for evaluating the underlying etiology and for surgical evaluation. CT can be used to assess for AR etiology, surgical planning, and coexisting coronary artery disease. CMR has already been established as a powerful imaging modality with reliable results and appears to be heading toward dominating the diagnostic approach to AR in the near future as the healthcare system catches up to its rapid emergence. While the role of aortography has diminished as other imaging modalities have increased, aortography remains valuable in assessing for PVL after valvular intervention.

Medical management is mainly limited to afterload reduction in AR with systemic hypertension. Invasive management, including SAVR or TAVR, is indicated based on severity, symptomatology, and LV dynamics. Significant recent innovations in AVR have been shown to improve outcomes to such a degree that many experts are considering pursuing valvular intervention even before the requirement by current guidelines. Additionally, advancements in THV devices continue to expand the boundaries of the transcatheter approach. The diagnosis and management of AR remains at the cutting edge of technology and innovation, and future research should endeavor to continue to drive this field forward.

## References

[1] Cowper W (1706). Of officiations or petrifications in the coats of arteries, particularly in the valves of the great artery. *Philosophical Transactions of the Royal Society of London*.

[2] Ravalli F, Kossar AP, Takayama H, Grau JB, Ferrari G (2020). Aortic Valve Regurgitation: Pathophysiology and Implications for Surgical Intervention in the Era of TAVR. *Structural Heart*.

[3] Mathieu P, Bossé Y, Huggins GS, Della Corte A, Pibarot P, Michelena HI (2015). The pathology and pathobiology of bicuspid aortic valve: State of the art and novel research perspectives. *The Journal of Pathology. Clinical Research*.

[4] Friedman T, Mani A, Elefteriades JA (2008). Bicuspid aortic valve: clinical approach and scientific review of a common clinical entity. *Expert Review of Cardiovascular Therapy*.

[5] Fernandes SM, Khairy P, Sanders SP, Colan SD (2007). Bicuspid aortic valve morphology and interventions in the young. *Journal of the American College of Cardiology*.

[6] Mai Z, Guan L, Mu Y (2021). Association between bicuspid aortic valve phenotype and patterns of valvular dysfunction: A meta-analysis. *Clinical Cardiology*.

[7] Tessler I, Albuisson J, Goudot G, Carmi S, Shpitzen S, Messas E (2021). Bicuspid Aortic Valve: Genetic and Clinical Insights. *Aorta*.

[8] Giusti B, Sticchi E, De Cario R, Magi A, Nistri S, Pepe G (2017). Genetic Bases of Bicuspid Aortic Valve: The Contribution of Traditional and High-Throughput Sequencing Approaches on Research and Diagnosis. *Frontiers in Physiology*.

[9] Li M, Kim JB, Sastry BKS, Chen M (2024). Infective endocarditis. *Lancet*.

[10] Moreillon P, Que YA, Bayer AS (2002). Pathogenesis of streptococcal and staphylococcal endocarditis. *Infectious Disease Clinics of North America*.

[11] Holland TL, Baddour LM, Bayer AS, Hoen B, Miro JM, Fowler VG (2016). Infective endocarditis. Nature Reviews. *Disease Primers*.

[12] Chasapi A, Mbonye KA, Bajomo O, Young WJ, Primus C, Ambekar S (2021). Clinical and echocardiographic predictors of decompensation in acute severe aortic regurgitation due to infective endocarditis. *Echocardiography*.

[13] Rodger L, Shah M, Shojaei E, Hosseini S, Koivu S, Silverman M (2019). Recurrent Endocarditis in Persons Who Inject Drugs. *Open Forum Infectious Diseases*.

[14] Rwebembera J, Nascimento BR, Minja NW, de Loizaga S, Aliku T, Dos Santos LPA (2022). Recent Advances in the Rheumatic Fever and Rheumatic Heart Disease Continuum. *Pathogens*.

[15] Dinkla K, Talay SR, Mörgelin M, Graham RMA, Rohde M, Nitsche-Schmitz DP (2009). Crucial role of the CB3-region of collagen IV in PARF-induced acute rheumatic fever. *PloS One*.

[16] Kim HR, Kim WK, Kim HJ, Kim JB, Jung SH, Choo SJ (2023). The fate of aortic valve after rheumatic mitral valve surgery. *The Journal of Thoracic and Cardiovascular Surgery*.

[17] Ho YC, Geng X, O’Donnell A, Ibarrola J, Fernandez-Celis A, Varshney R (2023). PROX1 Inhibits PDGF-B Expression to Prevent Myxomatous Degeneration of Heart Valves. *Circulation Research*.

[18] Sachdeva S, Desai R, Shamim S, Gandhi Z, Shrivastava A, Patel D (2021). Aortic valve myxoma-A systematic review of published cases. *International Journal of Clinical Practice*.

[19] Karmegaraj B (2024). Myxomatous degeneration of cardiac valves in a fetus with 6q25.1 (TAB2) deletion. *Cardiology in the Young*.

[20] Bernabeu E, Mestres CA, Loma-Osorio P, Josa M (2004). Acute aortic and mitral valve regurgitation following blunt chest trauma. *Interactive Cardiovascular and Thoracic Surgery*.

[21] Quintana EN, DeBose-Scarlett A, McLaren TA, Gondek SP, Smith MC, Alder MR (2024). Acute cardiogenic shock secondary to blunt traumatic aortic valve injury. *Trauma Case Reports*.

[22] Gelves J, Vasquez-Rodriguez JF, Medina HM, Marquez D, Jaimes C, Salazar G (2020). Severe Aortic and Tricuspid Valve Regurgitation after Blunt Chest Trauma: An Unusual Presentation. *CASE*.

[23] Evangelista A, Maldonado G, Gruosso D, Teixido G, Rodríguez-Palomares J, Eagle K (2016). Insights from the International Registry of Acute Aortic Dissection. *Global Cardiology Science & Practice*.

[24] Baliga RR, Nienaber CA, Bossone E, Oh JK, Isselbacher EM, Sechtem U (2014). The role of imaging in aortic dissection and related syndromes. *JACC. Cardiovascular Imaging*.

[25] Weigang E, Nienaber CA, Rehders TC, Ince H, Vahl CF, Beyersdorf F (2008). Management of patients with aortic dissection. *Deutsches Arzteblatt International*.

[26] Yuan SM, Jing H (2011). Cystic medial necrosis: pathological findings and clinical implications. *Revista Brasileira De Cirurgia Cardiovascular: Orgao Oficial Da Sociedade Brasileira De Cirurgia Cardiovascular*.

[27] Weldon CS, Ferguson TB, Ludbrook PA, McKnight RC (1977). A new operation for far-advanced cystic medial necrosis of the aortic root. *The Annals of Thoracic Surgery*.

[28] White NJ, Winearls CG, Smith R (1983). Cardiovascular abnormalities in osteogenesis imperfecta. *American Heart Journal*.

[29] Radunovic Z, Wekre LL, Diep LM, Steine K (2011). Cardiovascular abnormalities in adults with osteogenesis imperfecta. *American Heart Journal*.

[30] Covella M, Milan A, Totaro S, Cuspidi C, Re A, Rabbia F (2014). Echocardiographic aortic root dilatation in hypertensive patients: a systematic review and meta-analysis. *Journal of Hypertension*.

[31] Mulè G, Nardi E, Morreale M, Castiglia A, Geraci G, Altieri D (2017). The Relationship Between Aortic Root Size and Hypertension: An Unsolved Conundrum. *Advances in Experimental Medicine and Biology*.

[32] Zuo X, Liu L, Liu K, Zhang X, Ye R, Yang C (2023). Proximal aorta dilatation in hypertension. *Journal of Hypertension*.

[33] Gornik HL, Creager MA (2008). Aortitis. *Circulation*.

[34] Bley TA, François CJ, Abbara S, Kalva SP (2013). Chapter 25 - Inflammatory and Infectious Vascular Disorders. *Problem Solving in Cardiovascular Imaging*.

[35] Drago F, Merlo G, Rebora A, Parodi A (2018). Syphilitic aortitis and its complications in the modern era. *Giornale Italiano Di Dermatologia E Venereologia: Organo Ufficiale, Societa Italiana Di Dermatologia E Sifilografia*.

[36] Bekeredjian R, Grayburn PA (2005). Valvular heart disease: aortic regurgitation. *Circulation*.

[37] Brandt RR, Choi YH, Enriquez-Sarano M (2020). Chronic aortic regurgitation: diagnosis and therapy in the modern era. https://www.escardio.org/Journals/E-Journal-of-Cardiology-Practice/Volume-18/chronic-aortic-regurgitation-diagnosis-and-therapy-in-the-modern-era.

[38] Heidenreich PA, Hancock SL, Lee BK, Mariscal CS, Schnittger I (2003). Asymptomatic cardiac disease following mediastinal irradiation. *Journal of the American College of Cardiology*.

[39] Stewart JR, Fajardo LF (1984). Radiation-induced heart disease: an update. *Progress in Cardiovascular Diseases*.

[40] Bergfeldt L (1997). HLA-B27-associated cardiac disease. *Annals of Internal Medicine*.

[41] Bulkley BH, Roberts WC (1973). Ankylosing spondylitis and aortic regurgitation. Description of the characteristic cardiovascular lesion from study of eight necropsy patients. *Circulation*.

[42] Otto CM, Nishimura RA, Bonow RO, Carabello BA, Erwin JP, Gentile F (2021). 2020 ACC/AHA Guideline for the Management of Patients With Valvular Heart Disease: A Report of the American College of Cardiology/American Heart Association Joint Committee on Clinical Practice Guidelines. *Circulation*.

[43] Zoghbi WA, Adams D, Bonow RO, Enriquez-Sarano M, Foster E, Grayburn PA (2017). Recommendations for Noninvasive Evaluation of Native Valvular Regurgitation: A Report from the American Society of Echocardiography Developed in Collaboration with the Society for Cardiovascular Magnetic Resonance. *Journal of the American Society of Echocardiography*.

[44] Cape EG, Yoganathan AP, Weyman AE, Levine RA (1991). Adjacent solid boundaries alter the size of regurgitant jets on Doppler color flow maps. *Journal of the American College of Cardiology*.

[45] Teague SM, Heinsimer JA, Anderson JL, Sublett K, Olson EG, Voyles WF (1986). Quantification of aortic regurgitation utilizing continuous wave Doppler ultrasound. *Journal of the American College of Cardiology*.

[46] Stout KK, Verrier ED (2009). Acute valvular regurgitation. *Circulation*.

[47] Keane RR, Menon V, Cremer PC (2024). Acute Heart Valve Emergencies. *Cardiology Clinics*.

[48] Lancellotti P, Tribouilloy C, Hagendorff A, Popescu BA, Edvardsen T, Pierard LA (2013). Recommendations for the echocardiographic assessment of native valvular regurgitation: an executive summary from the European Association of Cardiovascular Imaging. *European Heart Journal. Cardiovascular Imaging*.

[49] Dujardin KS, Enriquez-Sarano M, Schaff HV, Bailey KR, Seward JB, Tajik AJ (1999). Mortality and morbidity of aortic regurgitation in clinical practice. A long-term follow-up study. *Circulation*.

[50] Mentias A, Feng K, Alashi A, Rodriguez LL, Gillinov AM, Johnston DR (2016). Long-Term Outcomes in Patients With Aortic Regurgitation and Preserved Left Ventricular Ejection Fraction. *Journal of the American College of Cardiology*.

[51] Detaint D, Messika-Zeitoun D, Maalouf J, Tribouilloy C, Mahoney DW, Tajik AJ (2008). Quantitative echocardiographic determinants of clinical outcome in asymptomatic patients with aortic regurgitation: a prospective study. *JACC. Cardiovascular Imaging*.

[52] Yang LT, Anand V, Zambito EI, Pellikka PA, Scott CG, Thapa P (2021). Association of Echocardiographic Left Ventricular End-Systolic Volume and Volume-Derived Ejection Fraction With Outcome in Asymptomatic Chronic Aortic Regurgitation. *JAMA Cardiology*.

[53] Yang LT, Michelena HI, Scott CG, Enriquez-Sarano M, Pislaru SV, Schaff HV (2019). Outcomes in Chronic Hemodynamically Significant Aortic Regurgitation and Limitations of Current Guidelines. *Journal of the American College of Cardiology*.

[54] Bonow RO, Lakatos E, Maron BJ, Epstein SE (1991). Serial long-term assessment of the natural history of asymptomatic patients with chronic aortic regurgitation and normal left ventricular systolic function. *Circulation*.

[55] Tornos MP, Olona M, Permanyer-Miralda G, Herrejon MP, Camprecios M, Evangelista A (1995). Clinical outcome of severe asymptomatic chronic aortic regurgitation: a long-term prospective follow-up study. *American Heart Journal*.

[56] Yang LT, Enriquez-Sarano M, Michelena HI, Nkomo VT, Scott CG, Bailey KR (2019). Predictors of Progression in Patients With Stage B Aortic Regurgitation. *Journal of the American College of Cardiology*.

[57] Vahanian A, Beyersdorf F, Praz F, Milojevic M, Baldus S, Bauersachs J (2022). 2021 ESC/EACTS Guidelines for the management of valvular heart disease. *European Heart Journal*.

[58] Cawley PJ, Hamilton-Craig C, Owens DS, Krieger EV, Strugnell WE, Mitsumori L (2013). Prospective comparison of valve regurgitation quantitation by cardiac magnetic resonance imaging and transthoracic echocardiography. *Circulation. Cardiovascular Imaging*.

[59] Willett DL, Hall SA, Jessen ME, Wait MA, Grayburn PA (2001). Assessment of aortic regurgitation by transesophageal color Doppler imaging of the vena contracta: validation against an intraoperative aortic flow probe. *Journal of the American College of Cardiology*.

[60] Shively BK, Gurule FT, Roldan CA, Leggett JH, Schiller NB (1991). Diagnostic value of transesophageal compared with transthoracic echocardiography in infective endocarditis. *Journal of the American College of Cardiology*.

[61] Fowler VG, Li J, Corey GR, Boley J, Marr KA, Gopal AK (1997). Role of echocardiography in evaluation of patients with Staphylococcus aureus bacteremia: experience in 103 patients. *Journal of the American College of Cardiology*.

[62] Shapiro SM, Young E, De Guzman S, Ward J, Chiu CY, Ginzton LE (1994). Transesophageal echocardiography in diagnosis of infective endocarditis. *Chest*.

[63] le Polain de Waroux JB, Pouleur AC, Goffinet C, Vancraeynest D, Van Dyck M, Robert A (2007). Functional anatomy of aortic regurgitation: accuracy, prediction of surgical repairability, and outcome implications of transesophageal echocardiography. *Circulation*.

[64] Siani A, Perone F, Costantini P, Rodolfi S, Muscogiuri G, Sironi S (2022). Aortic regurgitation: A multimodality approach. *Journal of Clinical Ultrasound*.

[65] Cawley PJ, Maki JH, Otto CM (2009). Cardiovascular magnetic resonance imaging for valvular heart disease: technique and validation. *Circulation*.

[66] Bolen MA, Popovic ZB, Rajiah P, Gabriel RS, Zurick AO, Lieber ML (2011). Cardiac MR assessment of aortic regurgitation: holodiastolic flow reversal in the descending aorta helps stratify severity. *Radiology*.

[67] Kammerlander AA, Wiesinger M, Duca F, Aschauer S, Binder C, Zotter Tufaro C (2019). Diagnostic and Prognostic Utility of Cardiac Magnetic Resonance Imaging in Aortic Regurgitation. *JACC. Cardiovascular Imaging*.

[68] Gelfand EV, Hughes S, Hauser TH, Yeon SB, Goepfert L, Kissinger KV (2006). Severity of mitral and aortic regurgitation as assessed by cardiovascular magnetic resonance: optimizing correlation with Doppler echocardiography. *Journal of Cardiovascular Magnetic Resonance: Official Journal of the Society for Cardiovascular Magnetic Resonance*.

[69] Faber M, Sonne C, Rosner S, Persch H, Reinhard W, Hendrich E (2021). Predicting the need of aortic valve surgery in patients with chronic aortic regurgitation: a comparison between cardiovascular magnetic resonance imaging and transthoracic echocardiography. *The International Journal of Cardiovascular Imaging*.

[70] Postigo A, Pérez-David E, Revilla A, Raquel LA, González-Mansilla A, Prieto-Arévalo R (2022). A comparison of the clinical efficacy of echocardiography and magnetic resonance for chronic aortic regurgitation. *European Heart Journal. Cardiovascular Imaging*.

[71] Attar R, Malahfji M, Angulo C, Nguyen DT, Graviss EA, Shah DJ (2025). Echocardiographic Evaluation of Chronic Aortic Regurgitation: Comparison With Cardiac Magnetic Resonance and Implications for Guideline Recommendations. *JACC. Cardiovascular Imaging*.

[72] Honda N, Machida K, Hashimoto M, Mamiya T, Takahashi T, Kamano T (1993). Aortic regurgitation: quantitation with MR imaging velocity mapping. *Radiology*.

[73] Dulce MC, Mostbeck GH, O’Sullivan M, Cheitlin M, Caputo GR, Higgins CB (1992). Severity of aortic regurgitation: interstudy reproducibility of measurements with velocity-encoded cine MR imaging. *Radiology*.

[74] Harris AW, Krieger EV, Kim M, Cawley PJ, Owens DS, Hamilton-Craig C (2017). Cardiac Magnetic Resonance Imaging Versus Transthoracic Echocardiography for Prediction of Outcomes in Chronic Aortic or Mitral Regurgitation. *The American Journal of Cardiology*.

[75] Capron T, Cautela J, Scemama U, Miola C, Bartoli A, Theron A (2020). Cardiac magnetic resonance assessment of left ventricular dilatation in chronic severe left-sided regurgitations: comparison with standard echocardiography. *Diagnostic and Interventional Imaging*.

[76] Gabriel RS, Renapurkar R, Bolen MA, Verhaert D, Leiber M, Flamm SD (2011). Comparison of severity of aortic regurgitation by cardiovascular magnetic resonance versus transthoracic echocardiography. *The American Journal of Cardiology*.

[77] Chatzimavroudis GP, Walker PG, Oshinski JN, Franch RH, Pettigrew RI, Yoganathan AP (1997). Slice location dependence of aortic regurgitation measurements with MR phase velocity mapping. *Magnetic Resonance in Medicine*.

[78] Borer JS, Truter S, Herrold EM, Falcone DJ, Pena M, Carter JN (2002). Myocardial fibrosis in chronic aortic regurgitation: molecular and cellular responses to volume overload. *Circulation*.

[79] Liu SK, Magid NR, Fox PR, Goldfine SM, Borer JS (1998). Fibrosis, myocyte degeneration and heart failure in chronic experimental aortic regurgitation. *Cardiology*.

[80] Taniguchi K, Kawamaoto T, Kuki S, Masai T, Mitsuno M, Nakano S (2000). Left ventricular myocardial remodeling and contractile state in chronic aortic regurgitation. *Clinical Cardiology*.

[81] Senapati A, Malahfji M, Debs D, Yang EY, Nguyen DT, Graviss EA (2021). Regional Replacement and Diffuse Interstitial Fibrosis in Aortic Regurgitation: Prognostic Implications From Cardiac Magnetic Resonance. *JACC. Cardiovascular Imaging*.

[82] Hashimoto G, Enriquez-Sarano M, Stanberry LI, Oh F, Wang M, Acosta K (2022). Association of Left Ventricular Remodeling Assessment by Cardiac Magnetic Resonance With Outcomes in Patients With Chronic Aortic Regurgitation. *JAMA Cardiology*.

[83] Haberka M, Starzak M, Smolka G, Wojakowski W, Gąsior Z (2024). Echocardiography and Cardiac Magnetic Resonance in the Assessment of Left-Ventricle Remodeling: Differences Implying Clinical Decision. *Journal of Clinical Medicine*.

[84] Myerson SG, d’Arcy J, Mohiaddin R, Greenwood JP, Karamitsos TD, Francis JM (2012). Aortic regurgitation quantification using cardiovascular magnetic resonance: association with clinical outcome. *Circulation*.

[85] Kočková R, Línková H, Hlubocká Z, Pravečková A, Polednová A, Súkupová L (2019). New Imaging Markers of Clinical Outcome in Asymptomatic Patients with Severe Aortic Regurgitation. *Journal of Clinical Medicine*.

[86] Merten C, Beurich HW, Zachow D, Mostafa AE, Geist V, Toelg R (2013). Aortic regurgitation and left ventricular remodeling after transcatheter aortic valve implantation: a serial cardiac magnetic resonance imaging study. *Circulation. Cardiovascular Interventions*.

[87] Rooijakkers MJP, Stens NA, van Wely MH, van der Wulp K, Rodwell L, Gehlmann H (2023). Diastolic delta best predicts paravalvular regurgitation after transcatheter aortic valve replacement as assessed by cardiac magnetic resonance: the APPOSE trial. *European Heart Journal. Cardiovascular Imaging*.

[88] Svensson LG, Adams DH, Bonow RO, Kouchoukos NT, Miller DC, O’Gara PT (2013). Aortic valve and ascending aorta guidelines for management and quality measures. *The Annals of Thoracic Surgery*.

[89] Hunt D, Baxley WA, Kennedy JW, Judge TP, Williams JE, Dodge HT (1973). Quantitative evaluation of cineaortography in the assessment of aortic regurgitation. *The American Journal of Cardiology*.

[90] Klein LW, Agarwal JB, Stets G, Rubinstein RI, Weintraub WS, Helfant RH (1986). Videodensitometric quantitation of aortic regurgitation by digital subtraction aortography using a computer-based method analyzing time-density curves. *The American Journal of Cardiology*.

[91] Schultz CJ, Slots TLB, Yong G, Aben JP, Van Mieghem N, Swaans M (2014). An objective and reproducible method for quantification of aortic regurgitation after TAVI. *EuroIntervention: Journal of EuroPCR in Collaboration with the Working Group on Interventional Cardiology of the European Society of Cardiology*.

[92] Tateishi H, Campos CM, Abdelghani M, Leite RS, Mangione JA, Bary L (2016). Video densitometric assessment of aortic regurgitation after transcatheter aortic valve implantation: results from the Brazilian TAVI registry. *EuroIntervention*.

[93] Abdelghani M, Tateishi H, Miyazaki Y, Cavalcante R, Soliman OII, Tijssen JG (2017). Angiographic assessment of aortic regurgitation by video-densitometry in the setting of TAVI: Echocardiographic and clinical correlates. *Catheterization and Cardiovascular Interventions*.

[94] Abdelghani M, Miyazaki Y, de Boer ES, Aben JP, van Sloun M, Suchecki T (2018). Videodensitometric quantification of paravalvular regurgitation of a transcatheter aortic valve: in vitro validation. *EuroIntervention*.

[95] Elder DHJ, Wei L, Szwejkowski BR, Libianto R, Nadir A, Pauriah M (2011). The impact of renin-angiotensin-aldosterone system blockade on heart failure outcomes and mortality in patients identified to have aortic regurgitation: a large population cohort study. *Journal of the American College of Cardiology*.

[96] Greenberg B, Massie B, Bristow JD, Cheitlin M, Siemienczuk D, Topic N (1988). Long-term vasodilator therapy of chronic aortic insufficiency. A randomized double-blinded, placebo-controlled clinical trial. *Circulation*.

[97] Schön HR, Dorn R, Barthel P, Schömig A (1994). Effects of 12 months quinapril therapy in asymptomatic patients with chronic aortic regurgitation. *The Journal of Heart Valve Disease*.

[98] Søndergaard L, Aldershvile J, Hildebrandt P, Kelbaek H, Ståhlberg F, Thomsen C (2000). Vasodilatation with felodipine in chronic asymptomatic aortic regurgitation. *American Heart Journal*.

[99] Greenberg BH, DeMots H, Murphy E, Rahimtoola SH (1981). Mechanism for improved cardiac performance with arteriolar dilators in aortic insufficiency. *Circulation*.

[100] Fioretti P, Benussi B, Scardi S, Klugmann S, Brower RW, Camerini F (1982). Afterload reduction with nifedipine in aortic insufficiency. *The American Journal of Cardiology*.

[101] Scognamiglio R, Fasoli G, Ponchia A, Dalla-Volta S (1990). Long-term nifedipine unloading therapy in asymptomatic patients with chronic severe aortic regurgitation. *Journal of the American College of Cardiology*.

[102] Sampat U, Varadarajan P, Turk R, Kamath A, Khandhar S, Pai RG (2009). Effect of beta-blocker therapy on survival in patients with severe aortic regurgitation results from a cohort of 756 patients. *Journal of the American College of Cardiology*.

[103] Evangelista A, Tornos P, Sambola A, Permanyer-Miralda G, Soler-Soler J (2005). Long-term vasodilator therapy in patients with severe aortic regurgitation. *The New England Journal of Medicine*.

[104] Barradas-Pires A, Merás P, Constantine A, Costola G, de la Cal TS, Rafiq I (2023). Repair of Aortic Regurgitation in Young Adults: Sooner Rather Than Later. *Journal of the American Heart Association*.

[105] de Meester C, Gerber BL, Vancraeynest D, Pouleur AC, Noirhomme P, Pasquet A (2019). Do Guideline-Based Indications Result in an Outcome Penalty for Patients With Severe Aortic Regurgitation?. *JACC. Cardiovascular Imaging*.

[106] Adam M, Tamm AR, Wienemann H, Unbehaun A, Klein C, Arnold M (2023). Transcatheter Aortic Valve Replacement for Isolated Aortic Regurgitation Using a New Self-Expanding TAVR System. *JACC. Cardiovascular Interventions*.

[107] Vahl TP, Thourani VH, Makkar RR, Hamid N, Khalique OK, Daniels D (2024). Transcatheter aortic valve implantation in patients with high-risk symptomatic native aortic regurgitation (ALIGN-AR): a prospective, multicentre, single-arm study. *Lancet*.

[108] Seiffert M, Diemert P, Koschyk D, Schirmer J, Conradi L, Schnabel R (2013). Transapical implantation of a second-generation transcatheter heart valve in patients with noncalcified aortic regurgitation. *JACC. Cardiovascular Interventions*.

[109] Seiffert M, Bader R, Kappert U, Rastan A, Krapf S, Bleiziffer S (2014). Initial German experience with transapical implantation of a second-generation transcatheter heart valve for the treatment of aortic regurgitation. *JACC. Cardiovascular Interventions*.

[110] Bashir H, Mendez-Hirata G, Simone AE, Garcia S, Kereiakes DJ (2024). Long-Term Outcomes and Durability of a Novel Dedicated Transcatheter Heart Valve to Treat Native Aortic Regurgitation. *JACC. Case Reports*.

[111] Wei L, Liu H, Zhu L, Yang Y, Zheng J, Guo K (2015). A New Transcatheter Aortic Valve Replacement System for Predominant Aortic Regurgitation Implantation of the J-Valve and Early Outcome. *JACC. Cardiovascular Interventions*.

[112] Liu H, Yang Y, Wang W, Zhu D, Wei L, Guo K (2018). Transapical transcatheter aortic valve replacement for aortic regurgitation with a second-generation heart valve. *The Journal of Thoracic and Cardiovascular Surgery*.

[113] Zhu L, Guo Y, Wang W, Liu H, Yang Y, Wei L (2018). Transapical transcatheter aortic valve replacement with a novel transcatheter aortic valve replacement system in high-risk patients with severe aortic valve diseases. *The Journal of Thoracic and Cardiovascular Surgery*.

[114] Samimi S, Hatab T, Kharsa C, Khan SU, Bou Chaaya RG, Qamar F (2025). Meta-Analysis of Dedicated vs Off-Label Transcatheter Devices for Native Aortic Regurgitation. *JACC. Cardiovascular Interventions*.

[115] Yin WH, Lee YT, Tsao TP, Lee KC, Hsiung MC, Wei J (2022). Outcomes of transcatheter aortic valve replacement for pure native aortic regurgitation with the use of newer- vs. early-generation devices. *Annals of Translational Medicine*.

[116] Sundt TM, Jneid H (2021). Guideline Update on Indications for Transcatheter Aortic Valve Implantation Based on the 2020 American College of Cardiology/American Heart Association Guidelines for Management of Valvular Heart Disease. *JAMA Cardiology*.

[117] Poletti E, De Backer O, Scotti A, Costa G, Bruno F, Fiorina C (2023). Transcatheter Aortic Valve Replacement for Pure Native Aortic Valve Regurgitation: The PANTHEON International Project. *JACC. Cardiovascular Interventions*.

[118] Zoghbi WA, Jone PN, Chamsi-Pasha MA, Chen T, Collins KA, Desai MY (2024). Guidelines for the Evaluation of Prosthetic Valve Function With Cardiovascular Imaging: A Report From the American Society of Echocardiography Developed in Collaboration With the Society for Cardiovascular Magnetic Resonance and the Society of Cardiovascular Computed Tomography. *Journal of the American Society of Echocardiography*.

